# Web-Based Human Papillomavirus Education and Professional Skills Intervention for Health Care Providers: Protocol for a Randomized Controlled Trial

**DOI:** 10.2196/60790

**Published:** 2025-04-03

**Authors:** Jacob Martinez, Jacquelin I Cordero, Meagan Whitney, Katie L LaRoche, Gabriel Frietze, Eva M Moya, Kristin Gosselink

**Affiliations:** 1 Border Biomedical Research Center College of Science The University of Texas at El Paso El Paso, TX United States; 2 College of Nursing The University of Texas at El Paso El Paso, TX United States; 3 Department of Health Promotion and Behavioral Sciences School of Public Health The University of Texas Health Science Center at Houston Houston, TX United States; 4 Department of Social Work College of Health Sciences The University of Texas at El Paso El Paso, TX United States; 5 Department of Physiology and Pathology Burrell College of Osteopathic Medicine Las Cruces, NM United States; 6 School of Pharmacy The University of Texas at El Paso El Paso, TX United States

**Keywords:** human papillomavirus, randomized controlled trial, HPV knowledge, HPV vaccine, health care provider, provider recommendations, communication strategies, Hispanic

## Abstract

**Background:**

The human papillomavirus (HPV) vaccine is an effective way to prevent HPV and its associated cancers. Provider recommendation has been shown to be one of the most successful strategies for increasing the uptake of the HPV vaccine; however, more training and resources are needed to help boost health care providers’ confidence and communication skills in recommending the HPV vaccine to their patients, particularly in underserved Hispanic communities where vaccination rates among all ages are lower.

**Objective:**

This study aims to compare HPV educational and professional skills intervention effectiveness on improving provider recommendations and patient communication strategies with health care providers serving the El Paso United States–Mexico border region.

**Methods:**

We will conduct a randomized, blinded, multiple posttest-only controlled behavioral trial using a parallel group design that will examine the effectiveness of a fully automated, web-based, culturally tailored HPV education and professional skills intervention containing unique reading material and video role-play, as compared to a standard Centers for Disease Control and Prevention fact sheet and video about general communication skills. Participants were recruited using a purposive sampling technique, both internet-based and in-person outreach events. Study data are being collected and managed using REDCap (Research Electronic Data Capture; Vanderbilt University) hosted at the University of Texas at El Paso. Chi-square analyses, ANOVA, and other statistical tests will be used with 2-tail α to reject null hypotheses at .05 to analyze the self-assessed outcome data. The Mauchly test of sphericity for each ANOVA and the Huynh-Feldt epsilon test or Greenhouse-Geisser correction to the degrees of freedom of the F-ratio will be reported for each significant effect. We may use multiple imputation procedures to handle the missing data (if applicable). This study is being conducted in the west Texas or southeast New Mexico region of the United States. Chi-square analyses will be used to assess associations between variables reported on the baseline provider knowledge, attitudes, and practice scales. We seek to examine self-assessed changes in provider attitudes and behaviors regarding HPV vaccine recommendation 1 month after receiving our unique multimedia and culturally tailored intervention.

**Results:**

Research and data collection for this clinical trial began in December 2023. Participant recruitment was closed by May 2024 (N=128), with final data collection expected to be completed by December 2024.

**Conclusions:**

This study team decided to report on the intervention protocol to help ensure transparency in the research process and facilitate the improvement of the research design. Tailored web-based educational programs for health care professionals, designed to address regional and patient population characteristics, may be a promising approach to enhancing the real-world implementation of clinical practice guidelines.

**Trial Registration:**

ClinicalTrials.gov NCT05120869; http://clinicaltrials.gov/ct2/show/NCT05120869

**International Registered Report Identifier (IRRID):**

PRR1-10.2196/60790

## Introduction

### Overview

The human papillomavirus (HPV) is a primarily sexually transmitted infection that affects approximately 40 million Americans and is associated with 6 types of cancer [1]. A total of 3 HPV vaccines (nonvalent, quadrivalent, and bivalent) have been developed, licensed, and shown to be effective in preventing high-risk HPV infection. Current immunization guidelines recommend that children aged 11-12 years initiate a 2-dose HPV vaccine series but can begin as early as 9 years of age. Young adults older than the age of 15 years with no previous history of HPV vaccination can start a 3-dose series; the 9-valent HPV vaccine is also approved to protect against 73% of HPV-associated cancers for adults older than 26 years of age [2]. Although national vaccination rates in the United States have steadily increased over the years, vaccination rates for adolescents remain well below the 80% designated target goal set forth by Healthy People 2030 [3]. The need for this vaccine is particularly salient considering the high direct and indirect costs of HPV and associated cancers in the United States; for example, Ong et al [4] found that the average cost of HPV-related cancer and other diseases was approximately US $38,056 per case.

### HPV Health Care Provider Recommendation Practices

Recent studies reveal that receiving a recommendation from a health care provider is a crucial factor influencing HPV vaccine uptake among parents of vaccine-eligible children and adults themselves [5-8]. However, in a study currently under review, many health care providers have reported that while they have sufficient knowledge and understanding about HPV and its treatment and prevention, they often lack the confidence to effectively communicate about HPV and recommend the vaccine to their patients. Similarly, some populations, such as adults, men, and sexual minority individuals (members of the LGBTQ+ [lesbian, gay, bisexual, transgender, and queer/questioning] community), are less likely to be encouraged to get vaccinated and tend to have inaccurate perceptions of the importance of vaccination [9]. Due to the need to improve training and resources for health care providers concerning HPV vaccine recommendation (in addition to the recommendation of other salient vaccines such as influenza and meningococcal vaccines [10,11]), researchers have explored the effectiveness of several approaches, theories, and foci when recommending HPV vaccination. The combined use of the presumptive approach (in which the provider assumes parents will vaccinate their child rather than communicating that the HPV vaccine is optional), along with motivational interviewing and a fact sheet for vaccine-hesitant clients, results in higher perceived levels of parental HPV vaccine acceptance [5]. Furthermore, studies [6,7] emphasize the importance of providing recommendations that “announce” the need for the HPV vaccine in a manner that is brief, easy to understand, and focused on cancer prevention.

Beyond the need for improved HPV vaccine recommendations among health care providers’ nationwide, enhancing communication strategies is particularly important for health care providers practicing with racial minority populations, such as predominantly Hispanic populations residing along the United States–Mexico border. Recent literature has identified both barriers and facilitators that exist in trying to increase HPV vaccine uptake in a diverse and predominantly Hispanic community [12]. Similarly, a recent quantitative study revealed that HPV vaccine acceptability in Hispanic young adults is associated with several factors, including the number of HPV informational sources, number of HPV discussions, health care provider recommendations, having a health care provider with similar characteristics, and family vaccine perceptions [12]. Another study found that culture-based factors such as familism and trusted sources of information can predict HPV vaccine acceptance [13]. Additionally, Xu et al [14] demonstrated that patient-provider health communication strategies aimed at enhancing HPV vaccine uptake among racial minority patients must be culturally tailored for the intended population, while including approaches that increase provider self-efficacy, use persistent language, reframing vaccine conversation focus from sex to cancer, and working with the community to culturally sensitize vaccine-related language. All these findings highlight the importance of receiving HPV vaccine recommendations from health care providers in a culturally competent manner, as well as the importance of family in shaping perceptions and behaviors related to vaccinations.

### Web-Based Health Care Professional Educational Interventions

To improve patient care and population health outcomes, health care providers rely on clinical practice guidelines (CPGs) to stay current with evidence-based recommendations [15]. However, the dissemination and implementation of guidelines, such as those for HPV vaccination and screening, vary across health professionals. While traditional print-based delivery methods for CPGs have largely shifted to digital platforms, digitizing guidelines on medical authority websites (eg, the Centers for Disease Control and Prevention [CDC]) have improved the access for health professionals. Despite improved access, time constraints often make it difficult for providers to actively search for and apply these guidelines without additional support. The absence of supportive modules that assist with real-world implementation, such as HPV recommendation practices, creates barriers to addressing gaps in care. Notably, research has shown that web-based medical educational modules can be as effective as in-person training in helping health care providers implement CPGs more efficiently [16].

### Intervention Theoretical Framework

Elements of the Health Belief Model have been examined as predictors of HPV vaccine acceptance and uptake among adults in the United States–Mexico border region in a previous study conducted by the researchers [13]. This model demonstrates that a broad range of intersectional factors play a role in an individual’s health choices and behaviors, such as perceived benefits (in this case, the belief that the HPV vaccine will prevent infection and cancer) and perceived severity, which have both been associated with HPV vaccine acceptance [13]. Frietze et al [13] also studied perceived safety and perceived harm as predictors of acceptance and uptake. Therefore, the proposed intervention has incorporated 4 tenets of the Health Belief Model (perceived benefits, severity, safety, and harm) into the intervention to increase provider vaccine recommendation practices and strategies. The tailored intervention also builds on educational models including HPV biology, epidemiology, and disease morbidity, coupled with role-played conversations in clinical video vignettes using the “announcement” approach, in which the health care provider works from the presumption that patients or parents will accept the vaccine. The research question is, will health care providers receiving a web-based, culturally tailored, HPV-specific professional skills intervention report stronger HPV prevention recommendation behaviors at 0-3-6 months post intervention, compared to health care providers receiving a web-based intervention using publicly available CDC HPV provider materials and general communication skills modules?

### Research Aims and Hypothesis

This study is guided by 2 aims. Aim 1 is to assess the knowledge, practices, and beliefs of emerging and current health care providers in the Paso del Norte Region related to HPV prevention, HPV vaccine, and HPV-associated cancers. Aim 2 is to facilitate the prevention of HPV-associated cancers in the Paso del Norte Region through strengthened recommendation of the HPV vaccine and screening by health care providers.

It is hypothesized that health care providers receiving a web-based, culturally tailored, HPV-specific professional skills intervention will report higher between-group changes in proportions on the HPV health care provider (HCP) practices scale at 0, 3, and 6 months post intervention, compared to health care providers receiving the standard web-based CDC HPV provider materials and communication skills active control intervention.

## Methods

### Ethical Considerations

This study was approved by the University of Texas at El Paso institutional review board (#1441487). Participation in the study was voluntary and confidential. All potential participants who were screened and met eligibility were invited to join the study and provided informed consent. Informed consent covered primary and secondary data collection procedures and data analysis and dissemination following the completion of the study. Participants consented to relinquish the ownership of anonymous data to the research team for dissemination (primary and secondary analysis). All study data will be deidentified and survey responses will be anonymous. Participants are compensated in the form of a US $30 electronic gift card for the completion of the initial survey and a US $10 electronic gift card for the completion of each of the follow-up surveys (US $50 total throughout the course of the study). The study information sheet made clear to participants that they were free to withdraw from the study at any time with no consequence to them, and withdrawal protocols with contact information were included.

### Study Trial Design

We will conduct a mixed (within-between) subjects longitudinal design in which participants are randomly assigned to 1 of the 2 conditions and will be assessed over 3 time points using a parallel group design to examine the effectiveness of a tailored versus a standard HPV education and professional skills intervention. Emerging and current health care providers will be randomly assigned to 1 of the 2 conditions. (1) Treatment condition in which they will receive a tailored Education and Professional Skills Intervention and (2) control condition in which they will receive general, publicly available (ie, CDC) information about HPV and communication skills. Participants will then be followed over 3- and 6-month time frames, and group differences will be assessed. Furthermore, this clinical trial protocol will follow the CONSORT-EHEALTH (Consolidated Standards of Reporting Trials of Electronic and Mobile Health Applications and Online Telehealth) to report the internet-based educational modules (behaviorally based treatment) [17]. The completed itemized checklist instrument is provided in Multimedia Appendix 1.

### Study Setting

The intervention is internet based, and participants will complete the survey using personally owned computers, tablets, or cell phones. This study is based on, deployed in, and will reach health care providers primarily in the Paso Del Norte Region, which includes El Paso County, Texas, and Southern New Mexico, including Dona Ana County, and the cities of Alamogordo, Hobbs, Artesia, Roswell, and Lovington. The population is predominantly Hispanic, with 82.9% (718,964/867,239) of the population of El Paso County, Texas, identifying as Hispanic or of Latin ancestry, and 50.2% (1,061,413/2,114,371) of the population of the State of New Mexico identifying as Hispanic or of Latin ancestry [18,19].

### Community Advisory Board

A community advisory board was formed, as community advisory boards have previously demonstrated to be effective when planning health-related programs and interventions to facilitate meaningful and structured engagement with local community members [20]. Our community advisory board consisted of a formalized collective of community members, research stakeholders, regional clinical partners, and other academics from several institutions, including The University of Texas at El Paso, Texas Tech University Health Sciences Center El Paso, and New Mexico State University. The community advisory board was instrumental in providing insight into the local community while providing insight to develop equitable partnerships with the community. The community advisory board has been involved in all phases of the intervention development, from the reconnaissance phase to the intervention’s development, and will remain a vital component of the intervention until the study has concluded and results are evaluated. To date, the community advisory board has met periodically and has been provided with a report of ongoing project-related activities and project progression. All community advisory board feedback and suggestions have been taken into consideration and retro fed back into project-related activities.

### Acceptability, Pretest, and Pilot

All components of the clinical trial (survey, control and experimental intervention modules, recruitment plan, and communications) underwent multiple rounds of review and revision by members of the research team. In addition, the intervention and survey were pilot-tested with a small group (n=7) of health care providers in training or in practice, and feedback from those individuals was incorporated into the final products. For example, demographic questions such as participant gender were changed to reflect participant’s concerns on gender identity (to include nonbinary and gender nonconforming individuals). For example, “Which of the following best describes your gender identity: male, female, transgender male, transgender female, gender variant/nonconforming, prefer not to answer,” and an “other” option which permits text entry. In addition, the pilot test helped to clarify questions regarding previous HPV infections: “How likely do you think it is that you have ever been infected with HPV?” and “How likely do you think it is that your current or recent partner(s) has (have) ever been infected with HPV?” A question was added to elicit the zip code in which the participant primarily practices or trains in, and the amount of time it would take to complete the study was adjusted.

### Consolidated Standards of Reporting Trials Flow Diagram

Progress through the phases of this planned randomized trial (ie, enrollment, intervention allocation, follow-up, and data analysis) will be visually represented using the CONSORT (Consolidated Standards of Reporting Trials) flow diagram [17]. A participant is considered to have completed the study if they have completed the initial postintervention assessment and the 3-month and 6-month follow-up assessments.

### Intervention Materials

The intervention materials and survey were developed and produced by members of the research team based on, and in alignment with, a health care provider survey and a community needs assessment conducted under the larger parent study [13,21]. HPV knowledge, practices, and beliefs will be assessed, as was done previously in formative research, with additional questions that further assess in-depth provider recommendation behaviors for HPV vaccination that is inclusive of vaccine-eligible age groups and genders. We will also inquire about provider comfort in communicating with patients about sexual health topics, vaccines, and cancer.

Participants will be randomly assigned to 1 of the 2 conditions: (1) the treatment condition, in which they receive the tailored HPV Education and Professional Skills Intervention, and (2) the control condition, in which they receive the General Education and Professional Skills Intervention. An internet-based platform will be developed to deploy the HPV Education and Professional Skills Intervention to emerging or current health care providers. The platform will be comprised of training modules divided into 3 sections paralleled among control and experimental groups—a document to be read, a video to be watched, and an activity to complete. Participants will take 15-30 minutes to complete all 3 sections of the module.

### Control Group Materials

The control group (active comparator) will receive the General HPV Education and Communication Skills Intervention materials, which will be comprised of a 3-page PDF document, a web-based video, and 4 rank-order multiple-choice questions to answer. The PDF document will be a standard, publicly available HPV fact sheet from the CDC. The video contains information on improving basic communication skills as would occur in a business or interpersonal setting. The multiple-choice questions will ask the participant to select their preferred mode of communication (ie, email, text, phone call, or face-to-face) in different scenarios.

### Experimental Group Materials

The experimental treatment group will receive the tailored HPV Education and Communication Skills Intervention materials, which comprised the same categories of content as in the control intervention. In this case, the 3-page PDF document is modeled after the CDC HPV fact sheet but includes data from the first phase of our program and informs the reader of important and relevant research findings from our region. The video was produced in-house and scripted to focus on improving provider communication with patients, specifically regarding HPV, the HPV vaccine, and addressing vaccine hesitancy. The multiple-choice questions are presented as clinical vignettes with response options that address patient concerns, and participants will rank them in terms of their perceived effectiveness.

Thus, the control module will engage participants to improve general communication skills and increase baseline knowledge about HPV, HPV vaccine, and HPV-associated cancers. The experimental intervention module, in contrast, will specifically engage participants to improve patient-provider communication and strengthen HPV vaccine recommendations through (1) identifying critical points of discussion regarding the HPV vaccine and patient health; (2) encouraging discourse on vaccines that are optional (eg, influenza) for patients; (3) sharing best practices for discussing vaccines with patients or parents who are vaccine-hesitant; (4) increasing provider understanding about generalized and region or culture-specific reasons for vaccine hesitancy among patients; and (6) facilitating increased knowledge about HPV, the vaccine, and its cancer relevance.

Surveys are to be delivered 3 and 6 months after the initial intervention, and the survey will again evaluate provider knowledge, practices, and beliefs regarding HPV, HPV vaccine, and cancer and will also specifically assess changes in provider behavior since the intervention. Uptake of the HPV vaccine by the provider or their children, assuming eligibility, will be tracked, as will provider recommendation behaviors and levels of comfort in interacting with patients when issues of sexual health, vaccine hesitancy, or cancer prevention arise.

### Study Outcome Measures

#### Provider Practices

The primary outcome of interest is between-group changes in proportions of provider practices. This will be measured on the 8-item HPV HCP practices scale (HCP vaccine recommendations, HCP screening recommendations, and HCP communication practices) scored on a Likert scale from never (1) to always (5). A composite score is developed that creates an average score for HCP vaccine recommendations, HCP screening recommendations, and HCP communication practices. Postintervention practices of health care providers will determine change in both strength and frequency of recommending the HPV vaccine to patients.

The secondary outcomes of interest include understanding provider beliefs and provider knowledge. These will be measured on previously validated scales adapted from the Vaccine Attitudes and Knowledge Survey (VAKS) [13,21].

#### Provider Beliefs

Provider beliefs (ie, attitudes toward HPV vaccine) will be measured using the 9-item HPV vaccine attitudes scale (perceived safety, perceived harm, and perceived effectiveness) scored on a Likert scale from strongly disagree (1) to strongly agree (5). A composite score is developed which creates an average score for perceived safety, perceived harm, and perceived effectiveness.

#### Provider Knowledge

Provider knowledge about HPV and the HPV vaccine will be measured using a previously adapted 14-item HPV knowledge scale scored as true (1) or false (0) [13,21]. A composite score is developed which creates an average score for 14 items and then multiplying by 100 to calculate a percentage.

### Participants

Participants will be recruited from the health care provider population in El Paso County, Texas, and Southern New Mexico. Both current (in practice) and emerging (in training) practitioners in the professions of medicine (ie, doctor of medicine, doctor of osteopathic medicine, physician assistant, nurse practitioner, pharmacy, or nursing) are eligible to participate in the study. To qualify, participants must be a current (in practice) or emerging (in training—student, resident, or fellow) health care provider, be between the ages of 18 and 65 years, be working, training, or living in the Paso del Norte Region (ie, El Paso County, TX-Southern New Mexico), have computer or internet literacy and access to an electronic device with internet access (eg, cell phone, tablet, and computer), and have the authorization to recommend the HPV vaccine. Participants will be excluded from the study if they are not affiliated with the El Paso United States–Mexico border region, have not previously participated in phases I or II of the larger parent research project, do not identify as a current or emerging health care provider, decline or are unable to participate in the full intervention and follow-up time points, or are unable to complete participation and activities in the English language.

### Recruitment

Recruitment took place over 6 months, from December 2023 to May 2024. No critical secular events fell into this study period. Participants who were eligible and agreed to participate in the study will be active in the project for 7 months post-initial recruitment. This time frame starts at the time of recruitment and ends at the final 6-month follow-up time point. Researchers used a purposive sampling technique to recruit participants through multimedia (ie, print and email) and professional network contacts. The study participants were recruited from (1) the main campus and some regional clinical hubs of the Burrell College of Osteopathic Medicine; (2) the University of Texas at El Paso College of Health Sciences, College of Nursing, and School of Pharmacy; (3) federally qualified health centers in the Paso del Norte Region; (4) private provider clinics; and (5) other health care workers and professional network connections that provide training and services to the Paso del Norte region residents. Special attention will be given to recruit practitioners in the specialties of pediatrics, family medicine, and obstetrics and gynecology due to their increased familiarity with vaccines, HPV, and cancer. All participation is entirely web-based. Once the participant is deemed eligible and agrees to participate, they will be provided with a study information sheet outlining that their participation is voluntary, and they can opt out at any time. Participants will be compensated for their time with a US $30 electronic gift card after completion of the initial training session and surveys (~30 minutes). Participants will receive email reminders to complete each of the 2 follow-up survey sessions at 3 and 6 months after completing the initial intervention, as well as an additional US $10 electronic gift card for participating in the follow-up survey sessions (~20 minutes per survey questionnaire).

### Randomization, Treatment Allocation, and Concealment

#### Sequence Generation

After determining eligibility, we will randomize participants on a 1:1 basis to intervention groups (experimental vs control) while controlling for the potential bias introduced by factors of sex, age, and career stage. To achieve this goal, we will use a randomization table within the REDCap (Research Electronic Data Capture; Vanderbilt University) platform to create balanced treatment groups based on these factors and reduce the effect of selection bias [22].

#### Concealment

This study is double masked (ie, participant and outcomes assessor). Participants will be randomly assigned to treatment and control groups; they will not be made aware of other participants’ receipt of treatment or control materials. Participants will be asked not to discuss this project outside of the intervention; delivery of the intervention or other educational materials will be internet-based, limiting the ability of participants to interact with one another during the study. Furthermore, to reduce bias in the implementation and assessment of the clinical trial (eg, outcome assessor), participant information and pre-post survey responses will be maintained separately from the assignment and delivery of the intervention.

### Measures

All the survey questionnaire measures or instruments will be made available electronically in English.

#### Screening Questionnaire

Participants completed a screening questionnaire delivered via the REDCap platform to determine their eligibility to participate in the study or intervention [22]. The questionnaire will assess items such as age, geographical location, and health care provider education status (ie, MD, DO, PA, NP, PharmD, or RN) of the potential participant. The questionnaire will also assess their comfort with auditory and visual content in the English language, and if they had prior participation in the earlier phase of this study. The screening questionnaire requires approximately 3 to 5 minutes to complete. Upon successful completion of the screening questions, participants will read and sign the consent form indicating that they agree to participate in the study.

#### Demographic and Background Questionnaire

The demographic and background questionnaire via the REDCap platform will gather demographic information (eg, age, gender, ethnicity, and income), as well as information about family composition, HPV vaccination status, trusted sources for medical information, general feelings about vaccines, trust in the government, and experiences with the recent COVID-19 endemic [22]. The demographic survey will only be administered at baseline prior to administering the intervention. The demographics survey requires approximately 5 to 8 minutes to complete.

#### Postassessment

A post-assessment via the REDCap platform will be administered at baseline (immediately following intervention), 3 months, and 6 months [22]. The post-assessment will include a modified version of the VAKS [13,21] to self-assess factors that influence levels of comfort in discussing HPV-related topics, patient-provider communication about HPV, vaccines, cancer risk, and additional questions designed to evaluate the impact of the interventions and the actual changes in practice in terms of vaccine uptake, patient communication, and vaccine recommendation behavior. Moreover, the outcome variables are included in the post-assessments such as the self-assessment of the proportions of provider practices (as indexed by the HCP practices scale), provider beliefs (as indexed by the HPV vaccine attitudes scale), and provider knowledge (as indexed by the HPV knowledge scale). These will be measured on previously published scales adapted from the VAKS [13,21]. In a previous study that administered the VAKS, Cronbach α ranged from 0.70 to 0.90 for the various scales included in the survey [13]. The post-assessment requires approximately 25-30 minutes to complete.

### Data Collection and Management

All study data will be collected using REDCap [22], a password-secured internet-based software application system developed to design and manage web-based databases and surveys, hosted at The University of Texas at El Paso. All data collected will be deidentified. REDCap will be maintained by the College of Science at The University of Texas at El Paso [22]. Data will remain stored for up to 5 years after completion of the study. Only the study team will have access to the data, as the team has received training on maintaining research data confidentiality, responsible conduct of research, and research ethics with human participants. Data will be accessed through password-protected and encrypted computers.

### Analytical Methods

All statistical analyses will be conducted using SPSS (version 26.0; IBM Corp) and R (version 4.0.3; R Core Team) and will use 2-tail α to reject null hypotheses at .05. Prior to conducting analyses, data will be screened to ensure that statistical assumptions are met and any transformations or adjustments to the model will be used. We may use multiple imputation procedures to handle missing data. Descriptive statistics will be reported for demographic variables including age, sex, gender, and health professional degree or field. A power analysis estimating repeated measures within-between interaction, assuming a small effect size (*f*=0.15), determined that approximately 110 total participants are required to provide an 80% chance of detecting an effect between the 2 experimental conditions (tailored intervention vs active control) across 3 time points (0, 3, and 6 months postintervention). To account for an estimated 10%-15% attrition, at least 100 participants needed to be recruited.

### Study Outcomes

The chi-square analyses will be used to assess associations between variables reported on the baseline provider knowledge, attitudes, and practice scales. Repeated measures mixed ANOVA with α set to .05 will examine whether change in our outcome variables (self-assessment of the proportions of provider practices, as indexed by the HCP practices scale), provider beliefs (as indexed by the HPV vaccine attitudes scale), and provider knowledge (as indexed by the HPV knowledge scale) is the result of the interaction between the experimental condition (tailored intervention vs active control) and time (at 3 time points) [23]. If no interaction emerges, we will conduct follow-up tests to determine whether any change in our outcome variables are due to one of the factors (experimental condition or time). We will calculate the Mauchly test of sphericity for each ANOVA, and the Huynh-Feldt epsilon test or Greenhouse-Geisser correction to the degrees of freedom of the *F*-ratio will be reported for each significant effect [23-25]. We may use multiple imputation procedures to handle missing data (if applicable).

### Future Directions and Plans for Dissemination

Plans for the dissemination of our findings include publication in peer-reviewed scholarly journals and presentation at scientific conferences, but we are also committed to sharing our data with the community that supports and contributes to this work. For both providers and patients, it is important to base medical decision-making on relevant and up-to-date information. Our findings will also be shared with health care providers in practice and in training at the institutions and locations in which our participants were included. Data will be reported in aggregate form, with no identifying information shared. This data may further be incorporated into the curriculum followed by providers in training in the Paso del Norte region and beyond.

## Results

### Overview

A larger parent grant (NIH/NIMHD 2U54MD007592) was funded in October 2019. Data collection of this subproject, a provider-focused randomized controlled trial (RCT), began in December 2023, with a total of 128 participants recruited by the closing of recruitment in May 2024. Primary data collection was completed in December 2024. As of March 2025, data analysis is in progress, with publication of results anticipated in winter 2025.

### Study Outcomes

#### Provider Practices

Results of the ANOVA model assessing the primary outcome of between-group changes in proportions of provider practices will be summarized and presented in tables and visuals.

#### Provider Beliefs and Provider Knowledge

Results of the ANOVA model assessing secondary outcomes of understanding provider beliefs and provider knowledge will be summarized and presented in tables and visuals.

## Discussion

### Principal Findings

The goal of this RCT was to develop and deploy an educational and professional skills intervention to increase health care provider HPV prevention recommendation scores. This is a critical factor in cancer prevention, and multiple factors contribute to the observation that, while HPV vaccine uptake in our region is higher than in many other regions, it still falls short of national targets. We anticipate finding that health care providers who receive our experimental intervention materials (culturally tailored videos specifically targeting effective HPV vaccine-related communication strategies) will demonstrate improved and more frequent HPV vaccination recommendation practices and behaviors with their patients, as compared to health care provider participants who receive the control materials (standard CDC-issued materials).

### Comparison to Prior Work and Strengths

Our approach to intervention with health care providers is not particularly novel, nor is the intention to increase provider vaccine recommendations since it has been well-established that provider recommendation is one of the most critical factors in patient decisions to get vaccinated [26]. What is innovative in our approach, however, is how we engage with and tailor skills-based training for providers in our region. The initial evaluation of provider knowledge, practices, and beliefs about HPV and the HPV vaccine informed us that HPV-related knowledge among providers is high. An intervention that provides additional education on HPV, the vaccine, and cancer was thus likely to have limited benefit and would not be a valuable use of provider time. In contrast, our intervention focuses on supporting the professional development of providers in the domains of modifying their own behavior and improving communication with patients. Increasing provider awareness of biases in vaccine recommendation and delivery, increasing their comfort in discussions about sexual health topics, and supplying them with tools for addressing patient hesitancy associated with vaccination were determined by our group to be more likely to produce the desired results.

A second way in which our research is innovative is to focus on emerging health care providers. Engaging with this group has the potential to produce multiple positive outcomes, including (1) increased vaccine uptake since many individuals in this group are vaccine-eligible but remain unvaccinated, (2) normalization of vaccine-related discussions in patient care if this becomes a consistent component of provider training, and (3) strengthened vaccine recommendations as the providers themselves serve as role models for vaccination to their patients. Third, we are committed to improving vaccine recommendation behavior by providers that serve a binational, majority-Hispanic population that is medically underserved and faces multiple barriers to vaccination. Previous work has shown that Hispanic women have high HPV vaccine acceptability [27], but this may vary based on age and birth origin [28].

### Limitations

This study recruited an internet population of health care providers in the United States–Mexico border region; therefore, findings may not directly translate to other health care provider populations nationally or globally. Furthermore, this intervention did not provide Continuing Medical Education (CME) credit, which would limit uptake outside of an RCT setting. Study participation involved a relatively large time commitment for providers in practice, which worked against recruitment and survey completion. We may, therefore, be somewhat limited in our ability to conduct subanalyses such as comparisons across provider specialties (eg, pediatrics vs family medicine) or other participant characteristics (eg, ethnicity or income level). As we have gathered data from health care providers in medicine, nursing, and pharmacy, different approaches to patient care in these professions may increase variability in the responses obtained. A pretest of HPV and vaccine-associated practices was not done in our study, and the use of solely self-reported data may be incomplete or may contain bias. Our random assignment of participants into control and experimental groups should protect against baseline group differences; however, we will still be able to determine the persistence of any changes in behavior regardless of participant starting point. If any group of providers demonstrates high vaccine recommendation rates at the onset of the study, it will be difficult to determine a statistically significant increase in behavior in response to the intervention. Still, identifying providers with strong recommendation practices will be of benefit to our project. Finally, we anticipate the loss of a limited number of participants through attrition over time for many possible reasons, perhaps reducing the power of our study and our data analysis capabilities at the 3- and 6-month time points.

### Conclusions

To improve population health, it is essential to look beyond interventions aimed solely at patient populations. Web-based educational programs for health care professionals are promising tools for enhancing the real-world implementation of CPGs. Tailoring these provider-level interventions to account for regional and patient population characteristics can also improve patient-provider communication as clinical guidelines evolve. Until HPV and other vaccine-preventable infections are eradicated, collaboration between health researchers and providers is critical to delivering accessible and effective professional development interventions. Furthermore, beyond reporting research findings of such studies, reporting specific intervention protocols helps ensure transparency in the research process and facilitates the improvement of population health interventions.

## Data Availability

The datasets generated or analyzed during this study are not publicly available as they are stored in the REDCap (Research Electronic Data Capture) repository maintained by the University of Texas at El Paso and subject to institutional access restrictions but are available from the corresponding author on reasonable request.

## Appendix

Multimedia Appendix 1 CONSORT-eHEALTH checklist (V 1.6.1).

Multimedia Appendix 2 Peer-review report from the External Advisory Committee for the National Institutes of Health (NIH) Research Centers in Minority Institutions (RCMI) Program - Border Biomedical Research Center at the University of Texas at El Paso (National Institutes of Health, USA).

## Abbreviations

CDC: Centers for Disease Control and Prevention
CME: Continuing Medical Education
CONSORT: Consolidated Standards of Reporting Trials
CONSORT-EHEALTH: Consolidated Standards of Reporting Trials of Electronic and Mobile Health Applications and Online Telehealth
CPG: clinical practice guideline
HCP: health care provider
HPV: human papillomavirus
LGBTQ+: lesbian, gay, bisexual, transgender, and queer/questioning
RCT: randomized controlled trial
REDCap: Research Electronic Data Capture
VAKS: Vaccine Attitudes and Knowledge Survey
