# Supplementary material for: Web-Based Human Papillomavirus Education and Professional Skills Intervention for Health Care Providers: Protocol for a Randomized Controlled Trial
Source: JMIR Res Protoc. 2025 Apr 3;14:e60790. doi: 10.2196/60790 (PMC12006767; doi:10.2196/60790)
Supplement: Multimedia Appendix 2 [file resprot_v14i1e60790_app2.pdf]

Border Biomedical Research Center University of Texas, El Paso

Report of the External Advisory Committee
November 29. 2023

Introduction

The External Advisory Committee (EAC) for the RCMI Program of the Border Biomedical Research Center (BBRC) at the University of Texas, El Paso, met on November 29, 2023. Two of the committee members attended in person and three attended via Microsoft *Teams*. A list of members of the Advisory Committee is provided in the BBRC Progress Report.

The P.I., Dr. Robert Kirken, and all key activity leaders of research projects and cores participated in the meeting. Dr. Kirken began the meeting with an overview of the program. Summaries of the aims and accomplishments of the three research projects (Basic, Clinical and Behavioral) were then presented by the project leaders followed by reports on the activities and progress over the past five years in four of the five cores (Community Engagement, Investigator Development, Recruitment and Research Infrastructure). Finally, extensive details for the five-year BBRC evaluation by UTEP Research Evaluation and Assessment Services (REAS) were presented All of this information is included in the progress report. The remainder of the meeting focused on the upcoming renewal submission.

The meeting was highly informative, with ample opportunity for interaction between the committee and the BBRC members. The AV setup for virtual attendees was excellent, making it possible to easily see and hear presenters and ask questions as desired. Substantial progress was demonstrated in numerous key areas as evidenced by the fact that the numbers of publications and conference presentations increased over the previous year. It is important to continue efforts to secure grant funding from governmental agencies such as the NIH and DOD in addition to setting up sponsor research agreements with companies.

As noted previously, but worth repeating, the accomplishments of the BBRC during the COVID-19 pandemic were astounding—notably that the BBRC was able to mobilize faculty and core facilities quickly to adapt to rapidly changing needs and limitations. The efforts and contributions of the BBRC ended up strengthening the importance of the Center in the community and provided opportunities for enhanced effectiveness.

Overall, the EAC considered the activities and progress of the BBRC to be outstanding and on the path to continuing as an exceptional and productive member of the RCMI community. (Accomplishments of the previous five years are briefly summarized in the corresponding sections below).

Administrative Core

Activities of the Administrative Core were not formally presented at the meeting, but could be appreciated in the context of the other BBRC components. This core is fundamental to the success of the entire BBRC/RCMI program. The core is responsible for a variety of activities that support all aspects of the program. Dr. Kirken is an able and experienced administrator and investigator. He provides coordinated management of the various components of the program and plays a prominent role in designing the current program. His guidance of the program through the past five years has enabled it to thrive despite extremely difficult circumstances. It is clear from the successes of the BBRC cores and projects that the Administrative Core is efficient and effective and provides the support that sustains the various components of the program. The staff members that assist this core are experienced and deeply committed to the success of the program as a whole.

Program evaluation is an important responsibility of the Administrative Core. As in previous years, the annual evaluation of the BBRC was carried out by the Center for Institutional Evaluation, Research, and Planning (CIERP) at UTEP. Specific findings of the 2023 CIERP evaluation of the program are included in the progress report and were also presented to the EAC at the meeting on November 29. Based on the information provided, the CIERP evaluations appear to be impartial and data-based. In addition to providing a compilation of BBRC activities, survey responses attest to the overall high level of satisfaction of BBRC members with the administrative and research infrastructure support available through the Center.

Community Engagement Core

The Aims of the Community Engagement Core (CEC), directed by Dr. Bibiana Mancera, are to: 1) create and foster meaningful partnerships to expand the breadth/scope of BBRC collaborations to facilitate community engaged research addressing expressed health-related needs and priorities; 2) leverage opportunities to engage, recruit, and retain participants in research studies within the predominantly Hispanic population of Mexican origin; and 3) augment culturally and linguistically sensitive dissemination strategies of research by partnering with community collaborators and stakeholders to reach the underserved and rural communities.

During the past year, the CEC has continued to increase efforts to foster formal collaborations with all healthcare systems/hospitals in El Paso. During COVID-19, the team was actively involved in vaccination efforts, vaccinating over 11,000 people at events they sponsored. They did an amazing outreach to the communities reaching over 210,000 people with information, and making over 500,000 social media posts. This ability to respond quickly to community needs has certainly put them in a better place to reach these populations with cancer information and for recruitment into clinical trials. Being able to respond to community needs is a great way to build trust and recognition. Much of this work was accomplished with the help of the trained community health workers (CHWs). Additionally, the team has added a CHW-led diabetes self-management education and support intervention (DSMES); 264 people have completed this educational program, and it is scheduled to be delivered to 900 diabetics.

Over the course of the grant, the CEC has received 13 Community-driven Organization Awards from the National Alliance for Hispanic Health for a total of $1,591,908 and a RADx-UP award for $307,000, 6 Paso del Norte Health/Community Foundation awards totally $622,722 and three awards from the University of New Mexico totaling $840,000. This is very impressive and shows that the core has used the funding wisely to leverage sources of money for the community.

The CEC has positioned itself to successfully support efforts conducted within the Hispanic community in El Paso and has demonstrated a growing knowledge and expertise to effectively assess and address negative health trends as well as significant changes within the population. The team has demonstrated and is working on developing health implementation models that can be replicated in other areas of the country with significant Latino populations. The CEC is an extremely active and successful core under the leadership of Dr. Mancera. The PI and her team are to be commended for continuing to seek and receive major funding for the CEC from several sources during 2023.

Investigator Development Core

The goal of the Investigator Development Core (IDC), directed by Dr. Marc Cox, is to support the efforts of BBRC investigators by enhancing their likelihood for success in securing research

funding, with emphasis on early-stage investigators (ESIs). Of particular importance is assistance in seeking extramural support for projects that address minority health and health disparities and fostering an environment that enhances career development for ESIs. This is accomplished through the following: effective mentoring of BBRC senior postdoctoral fellows and ESI faculty; supporting and incentivizing submission of extramural grant applications by ESIs; and providing funding opportunities to postdocs, ESIs, and senior PIs to obtain data for extramural grants, for example, through the Pilot Project Program.

The IDC has been very successful over the past five years. The core provided assistance for 87 opportunities for travel to conferences. Professional development opportunities focused on grant writing. For the NIH JUMPSTART @ UTEP program, there were 60 participants across five cohorts, representing six colleges and 20 departments, with the following outcomes: grant applications submitted by JUMPSTART participants, 374; total awarded to JUMPSTART participants as PI, $17.9M. The Pilot Project Program was also extremely successful: pilot projects, 13 awarded; total investment, $600,000; grant applications submitted by pilot project awardees, 19; funding secured by pilot awardees as a direct results of data from pilot projects, $1.7M; publications directly related to pilot projects, 18. There are plans to publish a paper about the UTEP program.

Recruitment Core

The primary goals of the Recruitment Core (RC), directed by Dr. Michael Kenney, are to identify, recruit, hire and maintain faculty who have the requisite knowledge and experience to enrich the research enterprise of the BBRC, consistent with the general research aims of the Center, especially in the area of health disparities.

The first original aim of the RC was the hiring of three clinical coordinators to execute processes essential for the acquisition of cancer patient biospecimens from collaborating hospitals. Unfortunately, the program had to be halted due to the pandemic and it has not been re-started. Other mechanisms are being used to obtain the specimens. The goal of Aim 2 was the hiring of established Investigators to lead health disparities research initiatives, develop collaborative research projects, enhance research competitiveness, and provide academic mentorship. There has been successful hiring of two new faculty members: Dr. Eda Koculi (College of Science, Department of Chemistry and Biochemistry) whose research focuses on the biochemistry of ribosome biogenesis; Dr. Yupeng Li (School of Pharmacy, Department of Pharmaceutical Sciences/ College of Science, Department of Chemistry and Biochemistry), whose expertise is in medicinal chemistry. The efforts of Aim 3 have been on targeted hiring in the data analytics field. This will expand the analytical capabilities of the UTEP BBRC, thereby providing the expertise for working with complex primary data sets associated with the new Center for Integrative and Translational Research. There was also success in this aim, with the following hires: Systems Analyst (Dr. Khodeza Begum), Statistical Analyst (Dr. Panfeng Liang), Machine Learning and Big Data Analyst (Dr. Cai Xu) and Computational Analyst - Multiomics (Dr. Jonathon Mohl). These hires represent a shift in vision and recognition of the importance of informatics and data analytics.

Research Infrastructure Core (RIC)

The BBRC Research Infrastructure Core (RIC) continues to provide critical support to the research projects that focus primarily on cancers of Hispanic origin. For example, the Biomolecule Analysis and Omics (BAO) and the Bioinformatics and Biostatistics (BBU) unit assisted Project 1 to discover mutations associated with the onset of Acute Lymphoblastic Lymphoma and other

types of cancers. Using the Oncominer software that was previously developed by the BBU, this unit has contributed to the rapid detection of single point polymorphisms (SNPs) in the genomes of human-derived tumor samples. The lead PI of Project 2, Dr. Zhang, has also used the RIC facilities to characterize diagnostic biomarkers in Hispanic patients with liver cancer (Ma, et al., Autoantibody against Tumor-Associated Antigens as Diagnostic Biomarkers in Hispanic Patients with Hepatocellular Carcinoma. Cells, 11, 3227; 2022). This and other work recently published by members of the BBRC is increasing the visibility of this Center and demonstrates its potential in improving health issues in the border region and Hispanic population. The RIC will further support health disparities research at UTEP and across all RCMI Institutions by working closely with the RCMI Coordinating Center.

Due to the increase in Covid-19 vaccinations and the drop in infections, all BBRC staff have been working regular schedules and assisting BBRC investigators on their projects since mid-2021. During this period, the main tissue culture facility was returned to normal use with the exception of two culture rooms that were used to isolate Covid-19 variants. After obtaining funds from the NIMHD RADx-UP ($1,036,464 for two years until 11/2022) as a supplement to the parent U54 grant, BBRC staff continued isolating and sequencing Covid-19 variants using the CCB and Genomic Analysis facilities. During this period, 3,600 samples were sequenced and both Delta and Omicron variants were detected with different lineages and sub-lineages within each variant. The trends of the evolution of the virus mirrored those of the rest of the country (Delta to Omicron BA.1, then BA.1 to BA.2, etc.; Robles-Escajeda, et al., manuscript in preparation).

The two research projects that are currently funded by the U54 grant involve studies that require live cells as well as genomic and proteomic data. Importantly, Project 2 includes efforts toward immortalization of primary culture cells to establish Hispanic cell lines focusing on prostate, hepatic, cervical/uterine, and hematological malignancies. As mentioned in the progress report, the CCB core also continues to characterize novel anti-cancer compounds; these studies have resulted in a number of publications and three patents (one provisional) by the core director and staff. The Director also reported that BBRC investigators published 65 articles in 2022, which was an increase of 21 publications from the previous year.

During the past years, a human tissue collection component was established within the Cellular Characterization and Biorepository (CCB) unit that has already collected 224 cancer tissue samples from Hispanic patients. Due to the increased interactions with local hospitals, the cancer tissue biorepository received 13 cancer and seven control tissues from local/regional medical collaborators during the past year. This is commendable, given that the BBRC does not have clinical coordinators to assist in sample collection. The efforts being undertaken to establish a potentially unique biorepository for tumor samples should be further expanded to generate cancer cell lines from the local population, which would be used in present and future studies.

The RIC's main mission is to support the projects funded by the U54 grant and this was evident in the presentations from both the project leads and the RIC director. Research Projects 1 and 2 (basic and clinical) have continued to benefit from access to the wide variety of established Core services, as well as new instrumentation that was purchased. As described in previous evaluations by this committee member, the RIC staff and cores not only assist the funded projects but also train a large number of undergraduate and graduate students. In terms of training and usage, the cores continued to train new staff and students. As reported by the RIC Director, the numbers of students and staff that were trained during the past year slightly decreased from 174 to 132; this was most likely due to the high numbers trained immediately after the pandemic in 2021 due to removal of in-person training restrictions. It is encouraging to see that mechanisms

are in place for the units to receive fees for services, and both the BAO and BBU were able to secure significant payments for services rendered. It is important to note that the evaluation of the RIC and the individual units reported by their internal evaluator (Dr. Carrejo and CIERP) revealed that the users of the cores are very satisfied with the services provided by the RIC. Overall, the RIC is doing an excellent job in maintaining efficient units devoted to increasing the research productivity of the U54 projects and the University.

Research Project 1 (Basic)
The Basic project ("Structure Function Relationship of Novel PhosphoRegulatory Sites and Effect of Acute Lymphoblastic Leukemia (ALL) SNPs on JAK3 Activity: Implications for New Cancer Treatments"), led by Dr. Georgialina Rodriguez, has an overall goal of developing better treatment strategies for JAK3-associated ALL, which may also hold therapeutic potential for other hematopoietic cancers by understanding how phospho-regulatory sites and SNPs promote an active kinase confirmation. This project is based on prior evidence of the role that the JAK3 protein may play in cancer, and other diseases as well. JAK3 harbors unique phosphoregulatory domains that are constitutively activated via conformational change caused by somatic mutations that can promote cancer. The project proposes that these phosphorylation sites are conserved in other JAKs and tyrosine kinase family members, and may contribute to progression of ALL. There is a critical need to better understand JAK3 to inform the development of novel inhibitors designed to target mutated JAK3 proteins. The project is highly appropriate for including in the BBRC, because studies have found that there is a higher incidence of leukemia and a higher rate of relapse among Hispanic children relative to their non-Hispanic counterparts.

There has been noteworthy progress made in this project, including discovery of 10 new phosphosites on JAK3 and identification of new mutations in Hispanic leukemia patients. It is now important to compare and contrast these findings in ALL from Hispanic origin with those of other racial/ethnic groups. Focus will also be expanded to analysis of other JAK family mutations. Structural studies of the mutant proteins are also underway, the results of which will be able to provide the basis for rational design of therapeutics such as small molecules inhibitors, which may have a superior therapeutic window compared to currently available drugs. As a results of the efforts in the Basic Project over the past five years, there have been 10 papers published and 25 conference presentations (13 undergraduates and 12 graduate students).

Research Project 2 (Clinical)
The Clinical Project ("Developing Non-Invasive, Cost-Effective and Accurate Screening for Prostate Cancer") is led by Dr. Wen-Yee Lee. The overarching aim of the project is to develop diagnostic and therapeutic strategies to reduce Hispanic cancer disparities using genetic, molecular, and physiological signatures obtained from Mexican-origin cancer patients, with a focus on prostate cancer. There are widely-recognized limitations in the current screening method for prostate cancer—determination of levels of prostate-specific antigen (PSA) and consequent conducting of biopsies of many millions of men where more than half end up with a negative result. The focus of the project has been on comprehensive analysis of urine samples as a way to assemble a biomarker panel that will be more sensitive and specific than the PSA test. The methodology is based on stir bar sorptive extraction (SBSE) followed by gas chromatography-mass spectrometry analysis. Informatics evaluation of the results have yielded exciting results that show promise for use as a diagnostic panel of urinary components that can serve as an inexpensive, non-invasive method for early detection of prostate cancer.

In addition to Dr. Lee, there have been two post-doctoral fellows and three graduate students working on the project. There have been nine papers published and four more under review in addition to 20 conference presentation (three invited, three at national meetings, six regional and 10 local). Particularly noteworthy is the fact that a U.S. patent has been obtained on the process (U.S. Patent No. 10,908, 162 B2; "Methods Related to Volatile Compounds in Genitourinary Cancers;" February 2, 2021) and a company has been founded for commercialization as a clinical application.

Research Project 3 (Behavioral)
The objective of this project (led by Dr. Eva Moya) is to assess HPV-related Knowledge, attitudes, and practices in a Majority-Hispanic community sample, identify barriers and facilitators of vaccine uptake, and utilize targeted interventions to improve vaccination, screening and health literacy across the lifespan. The project is an important component of the U54. It has the potential to reduce population-specific health barriers by understanding the barriers and by providing the information needed to change patients' perspectives on HPV vaccination, cancer screening and prevention, and overall early access to care, with the goal of early diagnosis/prevention of HPV-related cervical cancers.

The team has completed the community needs assessment (Phase 1). They found an association between having received the HPV Vaccine and gender identity, race, lifetime sexual activity and frequency of condom use. In addition, cultural factors influenced vaccine acceptance, as did the construct of the Health Belief Model. In Phase 2, the team developed a data collection tool for health professional skills assessment, which was tested with 93 health care providers. Findings from the survey of providers were that although they had a high level of knowledge, only about 50% were vaccinated, males are more likely to be unvaccinated, recommendations for vaccine favor females over makes, and provider recommendation is important. The team developed and piloted a health care provider educational intervention, and plan to deploy the intervention widely between December 2023 and February 2024. In Phase 3, the team developed and employed qualitative data collection tools, and completed 29 in-depth interviews with survivors and caregivers, three focus groups with community health workers, and social services providers. Using all the data collected, the team developed a bilingual community informed HPV prevention intervention and will pilot the intervention in December 2023. They intend to provide the intervention to 200 community members with follow up at three and six months in January 2024. The team has done excellent and meaningful work over the last year, and it is exciting to see how the professional and community interventions will increase HPV vaccine and decrease HPV related cancers.
